# Discovery and identification of *O*, *O*-diethyl *O*-(4-(5-phenyl-4, 5-dihydroisoxazol-3-yl) phenyl) phosphorothioate (XP-1408) as a novel mode of action of organophosphorus insecticides

**DOI:** 10.1038/s41598-017-03663-3

**Published:** 2017-06-15

**Authors:** Zhigang Zeng, Ying Yan, Bingfeng Wang, Niu Liu, Hanhong Xu

**Affiliations:** 1 State Key Laboratory for Conservation and Utilization of Subtropical Agro-bioresources, Key Laboratory of Natural Pesticide and Chemical Biology of the Ministry of Education, South China Agricultural University, Guangzhou, 510642 P.R. China; 2 School of Nuclear Technology and Chemistry & Biology, Hubei University of Science and Technology, Xianning, 437100 P.R. China

## Abstract

Organophosphorus (OP) insecticides play an important role in pest control. Many OP insecticides have been removed from the market because of their high toxicity to humans. We designed and synthesized a new OP insecticide with the goal of providing a low cost, and less toxic insecticide. The mode of action of *O*, *O*-diethyl *O*-(4-(5-phenyl-4, 5-dihydroisoxazol-3-yl) phenyl) phosphorothioate (XP-1408) was studied in *Drosophila melanogaster*. Bioassays showed that XP-1408 at a concentration of 50 mg/L delayed larval development. Molecular docking into *Drosophila* acetylcholinesterase (AChE) and voltage-gated sodium channels suggested that XP-1408 fitted into their active sites and could be inhibitory. Whole-cell patch clamp recordings indicated that XP-1408 exhibited synergistic effects involving the inhibition of cholinergic synaptic transmission and blockage of voltage-gated potassium (K_v_) channels and sodium (Na_v_) channels. In conclusion, the multiple actions of XP-1408 rendered it as a lead compound for formulating OP insecticides with a novel mode of action.

## Introduction

Organophosphorus (OP) insecticides are the ones among classic pesticides. To date, OP insecticides have shown the main functions in pest control, due to their low cost, high performance, broad spectrum and some other advantages. However, their negative issues such as increasing insecticide resistance, risk to human health, environmental concerns and so on, have reduced their own market^1^. Therefore, discovering novel OP insecticides with low toxic and multi-target effects is of great significance and has gained a long term interest.

OP insecticides bear a diverse group of chemical structures that exhibit a wide range of physicochemical properties, with their primary toxicological action arising from inhibition of acetylcholinesterase (AChE)^2^. The typical symptom of acute OP insecticides toxication is neural hyperexcitation followed by convulsion, paralyses and death. AChE inhibition and excessive accumulation of acetylcholine (ACh) in cholinergic synapses have been proposed as the primary mechanism of hyperexcitation induction by OP insecticides^3, 4^. However, there are evidences that OP insecticides also interact with targets other than AChE of invertebrate and vertebrate, which could potentially affect the neuronal excitability *via* modulating the membrane ionic channel currents. For instance, some OP insecticides interact directly with receptors of the cholinergic system or modulate the receptor expression level^5, 6^, block chloride channels in *Aplysia* neurons, modulate calcium and calcium dependent potassium channels in snail neurons, and induce apoptosis by impacting on mitochondria^7–9^.

In complex multicellular organisms, many physiological processes (e.g., growth and development) require rapid and accurate transmission of information among cells and tissues, and tight coordination of distinct functions. The processes that are controlled by electrical signals, are mediated partly by members of the voltage-gated ion channel protein superfamily^10^. For instance, voltage-gated sodium (Na_v_) channels are transmembrane proteins responsible for action potential (AP) initiation and propagation in excitable cells, including nerve, muscle and neuroendocrine cells^11^. Voltage-gated potassium (K _v_) channels are widely expressed in the central and peripheral nervous system and are crucial mediators of a wide variety of physiological processes, including cell excitability, the maintenance of resting membrane potential, neurotransmitter release, signal transduction and cell proliferation^12, 13^. K _v_ channels have many types and the specific roles played by individual K channel types can be elucidated by taking advantage of differences in voltage-dependence, kinetics, and pharmacological specificity^14^. Sodium and potassium channels of mammalian and insect have similar functional characteristics in physiology and pharmacology, whereas tiny differences in the properties are still helpful to distinguish different isoforms and contribute to their specialized functional roles. And, many ion channel toxins, especially those targeting Na_v_ channels, are useful insecticidal compounds, because of their rapid lethal activity, high potency, and relative insect selectivity. However, with an increasing incidence of insecticide resistance, there is an eagerness to explore novel targets, such as insect K _v_ and voltage-activated calcium (Ca_v_) channels^15–17^.

Acetylcholine is the major excitatory neurotransmitter in the insect central nervous system (CNS), and nicotinic acetylcholine receptors (nAChRs) mediate the predominant form of fast excitatory synaptic transmission and represent a major target for several insecticides^18, 19^. The antennal lobe projection neurons (PNs) of *Drosophila* are not only the most cholinergic but also receive cholinergic synaptic input, and fire spontaneously^20^. It makes *Drosophila* whole-brain preparation as an ideal intact neural model to investigate the influences of chemical compounds on neural functions of insect CNS^21^.

Having the above described considerations in mind, we are interested in developing novel OP insecticides with targeting at new sites in insect nerve system. And inspired by the discovery of mode of action of isoxazoline insecticides^22, 23^, we designed and synthesized two kinds of novel phosphorothioate and phosphoramidothioate derivatives containing diphenyl isoxazoline. Although their insecticidal activities against target pests (e.g., *Spodoptera litura*, *Plutella xylostella*, etc.) were not ideal at their assigned concentration, we found an interesting phenomenon. The development of insects was delayed by the derivatives, especially with the compound *O*, *O*-diethyl *O*-(4-(5-phenyl-4, 5-dihydroisoxazol-3-yl) phenyl) phosphorothioate (XP-1408). Thus, to identify the potential mode of action, we herein report the possible physiological targets of XP-1408 by using molecular docking and whole-cell patch clamp approaches. The results show that the mode of action of compound XP-1408 is due to the synergistic actions of the suppression of cholinergic synaptic transmission, the blockage of K _v_ and Na_v_ channels, and AChE inhibitoion.

## Results and Discussion

### Chemical synthesis

**Figure 1:**
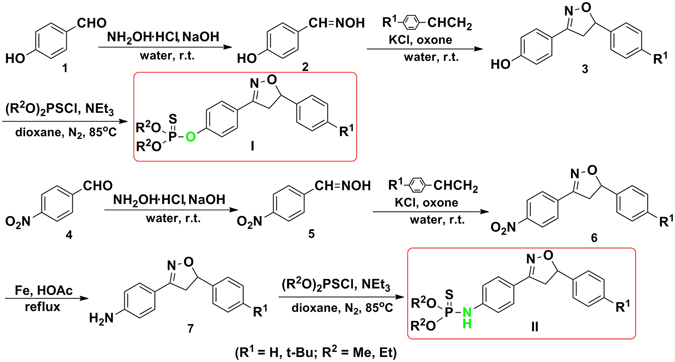
Procedures for Synthesis of Target Compounds I and II.

Compounds I and II were obtained based on its synthetic feasibility and were synthesized successfully according to the procedures as shown in Fig. 1. The reaction conditions of the final product were optimized by using the synthesis of compound I-2 as a model reaction. The synthesis was carried out in different solvents, such as dioxane, acetonitrile, toluene, tetrahydrofuran (THF) and *N*, *N*-dimethylformamide (DMF). The solvent effect is summarized in Table 1. Reaction in pyridine did not form any desired product. In solvents such as DMF, acetonitrile and toluene, only traces of product were formed. This observation led us to try a middle polarity solvent to activate the reaction substrates for the further optimization. Indeed, middle polarity solvents such as THF allowed moderate product formation of 25%. And a maximum yield of up to 66% was achieved when the reaction was performed in dioxane for 12 h. It might be speculated that middle polarity solvents such as some ethers are beneficial to accomplish the reaction. The structures of all the target compounds were determined by (^1^H/^13^C/^31^P) NMR, mass or high-resolution mass measurement (HRMS). The main characteristic of XP-1408 in ^1^H NMR spectra is the presence of double doublet at δ H 5.74–5.70 ppm, 3.76–3.70 ppm and 3.34–3.29 ppm, corresponding to the 5- and 4-position hydrogen protons of isoxazoline ring, respectively. The spectroscopic data of ^13^C NMR spectra, ^31^P NMR spectra and MS (HRMS) are also consistent with the structures of title compounds. Therefore, the obtained spectroscopic data are in accordance with their assigned structures, confirming the successful synthesis of title compounds.

**Table 1:** Effects of Different Solvents for Synthesis of Compound I-2.

No.	Solvent	Temp (°C)	Acid-binding agent	Time (h)	Yield (%)
1	pyridine	85	/	24	No reaction
2	acetonitrile	reflux	NEt_3_	96	Trace
3	toluene	85	NEt_3_	48	Trace
4	DMF	85	NEt_3_	48	Trace
5	THF	reflux	NEt_3_	24	25%
6	dioxane	85	NEt_3_	12	66%

### Bioassay of insecticidal activity

The insecticidal activity of all the target compounds was studied. At a concentration of 100 mg/L, both compounds I (arylisoxazoline-phosphorothioates) and compounds II (arylisoxazoline-phosphoramidothioates) have no insecticidal activity against the 2nd-instar larvae of *Spodoptera litura* in 72 h, while the insecticidal activity of dimethoate and phoxim were 100%. However, it is found that the developmental period of *Spodoptera litura* treated with compounds arylisoxazoline-phosphorothioates has been extended in the following two weeks. The pupation rate was between 55–80% (Table 2), while the other ones treated with arylisoxazoline-phosphoramidothioates did not have such a correlation. Therefore, we speculated that the development of the target insects may be delayed by the phosphorothioates. Additionally, compound I-2 (XP-1408) exhibited good bioactivity (pupation rate 50%) as mentioned. Next, we chose it for more detailed experiments to identify the possible novel bioactivity.

**Table 2:** Pupation rate of *S*. *litura* after treatment for 14 days*.

Compd.	R^1^	R^2^	Pupation Rate (%)	Compd.	R^1^	R^2^	Pupation Rate (%)
I-1	H	Me	73c	II-1	H	Me	95a
I-2	H	Et	55d	II-2	H	Et	96a
I-3	t-Bu	Me	80b	II-3	t-Bu	Me	98a
I-4	t-Bu	Et	69c	II-4	t-Bu	Et	93a
Dimethylsulfoxide (DMSO)			98a				

^*^From 5 replicates, each replicate contained 20 test insects. Data followed by the different letters means significant difference (*P* < 0.05, method of Duncan’s Multiple Range Test). The pupation rate is calculated using the following formula: pupation rate (%) = (1 – *n*/*N*) × 100, where *n* is the number of pupated insects, *N* is the total number of test insects.

### Insect development experiment for XP-1408

**Figure 2:**
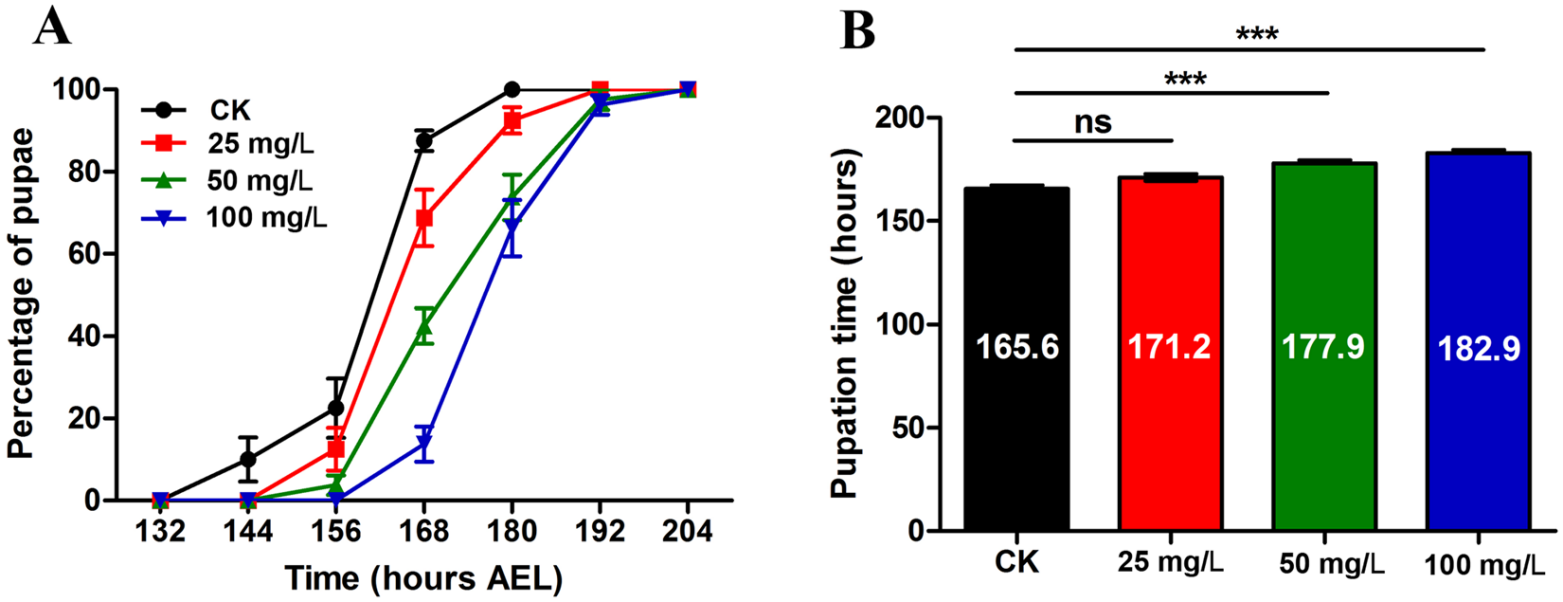
Effects of XP-1408 on pupariation of the third instar *Drosophila* larvae. (**A**) The larvae developmental curves were drawn based on the pupation in different time points after treated with 25, 50 and 100 mg/L of XP-1408. n = 20 larvae, four repetitions. (**B**) The total pupation time was counted from the data in CK and XP-1408 groups with different concentration. ***P < 0.001vs. CK. ns, not significant.

The growth inhibition activity of XP-1408 was investigated by using *Drosophila*, a model insect commonly used in the development experiment. The development analysis could be interfered by the different development stages of *Drosophila* larvae. Therefore, a rigorous experimental protocol was employed. Synchronized *Drosophila* larvae at 72 h after egg laying (AEL) were exposed to 25, 50, and 100 ppm of XP-1408 and the development time of larvae was monitored in 12 h intervals. Both 50 and 100 ppm of XP-1408 significantly delayed the pupation time of larvae by approximately 12 and 17 h, respectively (Fig. 2A and B). These results indicated that XP-1408 had a strong growth inhibition activity against *Drosophila* by inducing robust developmental delays in the larva-to-pupa transition.

### Computational simulations

**Figure 3:**
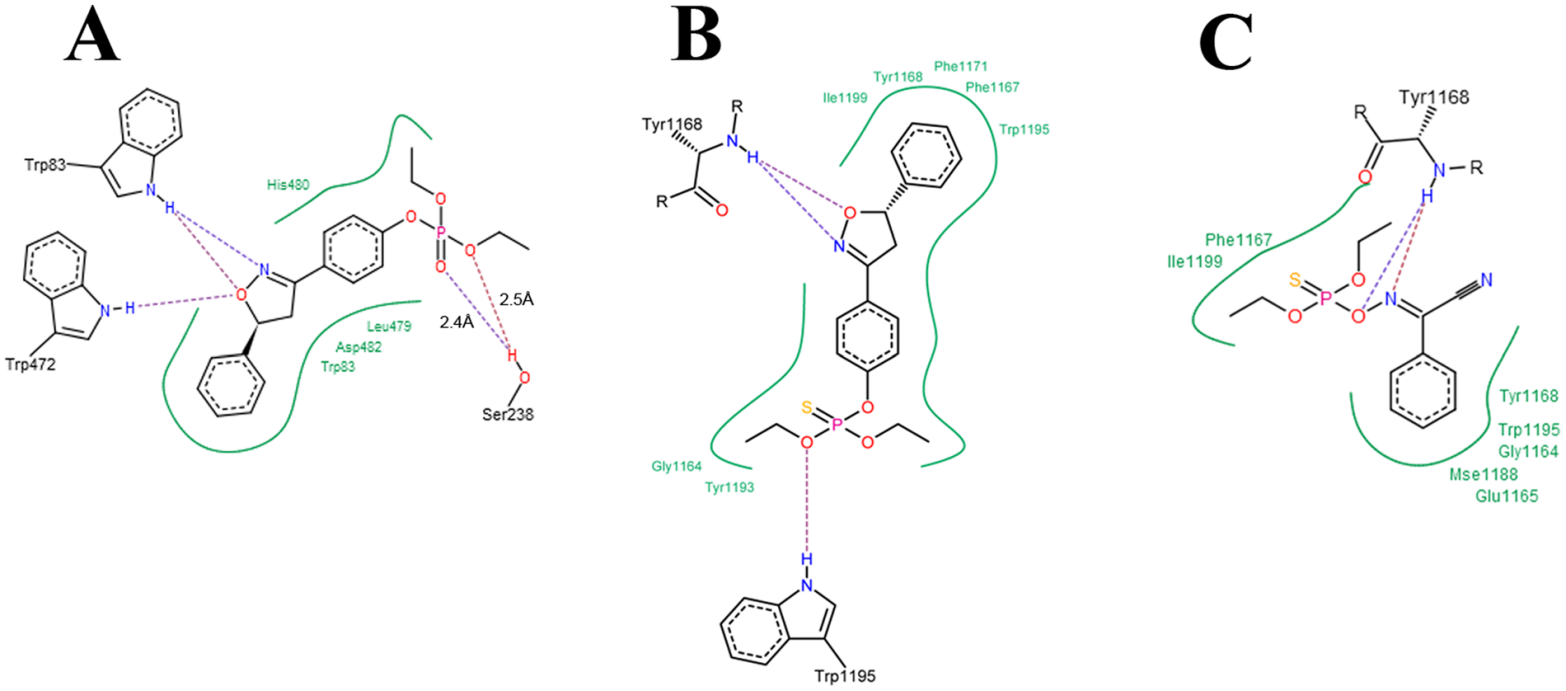
The 2D interaction modes of XP-1408 and phoxim within the binding site of corresponding LBD. (**A**) Docking model of oxidative desulfurated XP-1408 combined with AChE. (**B**) Interaction mode of XP-1408 with Na_v_ channel. (**C**) Interaction mode of phoxim with Na_v_ channel.

To search and clarify the binding mode of XP-1408, the compounds were docked into the active site of the AChE and Na_v_ channel. It is reported that water played a catalytic assistance role in the reactivity of OP’s in the active site of AChE^24^. Currently, water molecules were deleted, due to the primary investigation for the interaction between the target compound and the amino acid residues and the restriction of the semi-flexible docking simulation. According to the metabolic pathway *in vivo* of phosphorothioate insecticides, oxidative desulfurated XP-1408 was docked into *Drosophila* AChE. The results showed that classical residues (e.g., Ser238 and His480) in the ligand binding domain (LBD) were involved in the interactions between oxidative desulfurated XP-1408 and AChE (Fig. 3A). Furthermore, oxidative desulfurated XP-1408 could form hydrogen bonds with the residues in the LBD. For instance, there were two strong hydrogen bonds forming between the hydrogen atom of Serine OH and the oxygen atom of phosphate ester group, and the distance was 2.4 Å and 2.5 Å, respectively. Meanwhile, the native ligand Ach was also docked into the active site of AChE under the same conditions (Figure shown in supplementary). The docking score was −11.54, much higher than that of oxidative desulfurated XP-1408 (−20.78). It revealed that the binding energy between oxidative desulfurated XP-1408 and AChE was much stronger than that of Ach. These results suggested that the interactions between XP-1408 and AChE were similar to that of commercial phosphorothioate insecticides. On the other hand, the binding site of XP-1408 on Na_v_ channel was located in the groove between two subunits (Fig. 3B), which was similar to phoxim (Fig. 3C). However, the interactions between XP-1408 and Na_v_ channel were hydrogen bonds and hydrophobic electro-static interactions, indicating that the binding mode of XP-1408 differed from the control OP insecticide phoxim. Even more important was the observation that the docking score of XP-1408 (−15.9198) against the Na_v_ channel was much lower, compared to the reference phoxim (−6.8522). It meant that the adhesion force between XP-1408 and the Na_v_ channel was much stronger than that of phoxim. So, it is reasonable to speculate that Na_v_ channel is the extra binding sites of compound XP-1408, besides of AChE.

### Effects of XP-1408 on the membrane excitability in PNs of *Drosophila* brain

**Figure 4:**
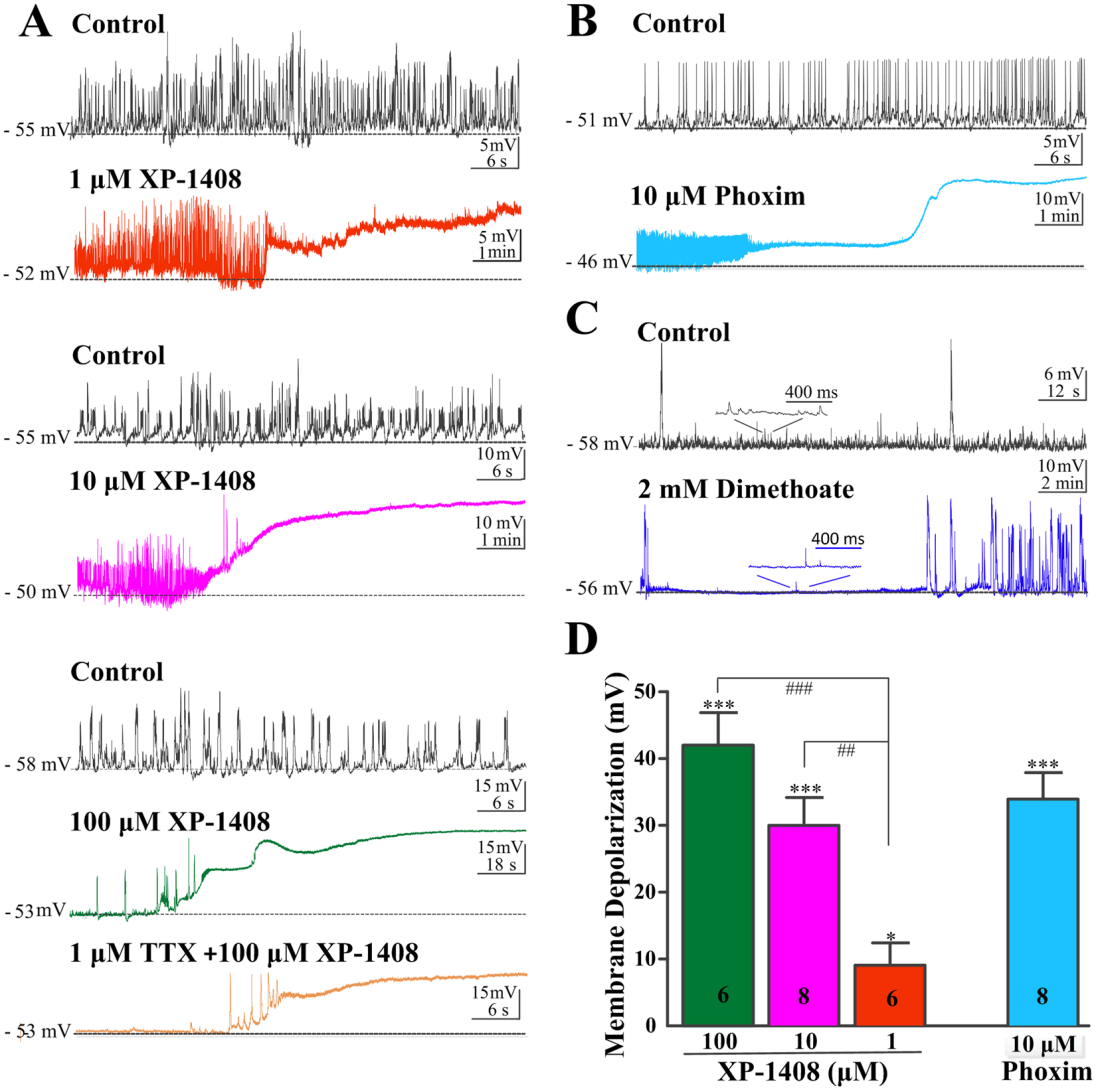
Effects of XP-1408 on spontaneous activities and resting membrane potentials in *Drosophila* PNs. (**A**) Representative voltage traces of individual PNs exposed to different XP-1408 concentrations (bath application). In the presence of XP-1408, a rapid depolarization was seen accompanied with activation of a high frequency burst of spontaneous activities, followed by inhibition of all activities. Preincubation of 1 μM TTX only partially suppressed the depolarization induced by XP-1408. (**B**) Current clamp recordings before (upper trace) and 10 min after application of phoxim (10 μM) (bottom trace) from PNs. (**C**) Current clamp recordings before and 10 min after application of dimethoate (2 mM) from PNs. Inset, the high time resolution of the indicated region. (**D**) Population data summarizing the peak amplitude of the depolarization induced by XP-1408 and phoxim. XP-1408 mediated depolarization was obvious in the range of 1–100 μM concentration, and significantly differed between low concentration (1 μM) and high concentration (10 μM and 100 μM). There was no significant difference in depolarization amplitude between XP-1408 and phoxim at 10 μM concentration. Each bar indicates the means ± SEM from indicated neurons (n).

Given that the typical toxic symptom is neural hyperexcitability followed by inhibition of nerve conduction after acute exposure to OP insecticides. Therefore, initial experiments were designed in order to understand whether XP-1408 has central neurotoxicity similar to that of analogue OP insecticides. Addition of XP-1408 (10 μM) caused a robust depolarization of resting membrane potentials (RMP) and an increase in spontaneous activities in the early stage (including AP and postsynaptic potential) which then became blocked. The characteristic of depolarization had two phases. The initial phase showed a stronger depolarization to a peak, and then followed by a sustained depolarization. We next investigated the concentration dependence of XP-1408-mediated actions. Over the range tested (1–100 μM), XP-1408 produced a concentration-dependent increase of the depolarization magnitude, and the onset of depolarization typically appeared within 1–10 min after the treatment. The effects of XP-1408 on the depolarization of RMP could not completely be suppressed by the preincubation of 1 μM of tetrodotoxin (TTX, Fig. 4A). The peak amplitude of XP-1408-mediated depolarization was obvious and averaged 8.9 ± 2.3 (*P < 0.05, n = 6), 29.4 ± 3.8 (***P < 0.001, n = 8) and 42.02 ± 4.9 mV (***P < 0.001, n = 6) at 1 μM, 10 μM and 100 μM, respectively. Overall, XP-1408-mediated depolarization significantly differed between the low concentration (1 μM) and high concentrations (10 μM, ^##^ *P* < 0.01; 100 μM, ^###^ *P* < 0.001, ANOVA with Bonferroni multiple comparisons) (Fig. 4D).

In addition, we further investigated the excitatory actions of another two representative OP insecticides, phoxim and dimethoate. Similar to XP-1408, phoxim also produced a concentration-dependent depolarization of PNs and the extents of depolarization reduced by 10 μM phoxim was conspicuous as well (***P < 0.001, n = 8) (Fig. 4B and D), whereas the dimethoate treatment had no effect on RMP and only temporarily inhibited the spontaneous activities at a high concentration (2 mM) (Fig. 4C). The peak depolarization amplitude of 10 μΜ of phoxim had no significant difference compared to that of 10 μM of XP-1408 (XP-1408, 29.4 ± 3.8 mV vs. phoxim, 33.3 ± 3.5 mV; *P* > 0.05. *t*-test) (Fig. 4D). The results of the initial experiment indicated that XP-1408 may have multiple targets.

### Effects of XP-1408 on cholinergic synaptic transmission in *Drosophila* brain

**Figure 5:**
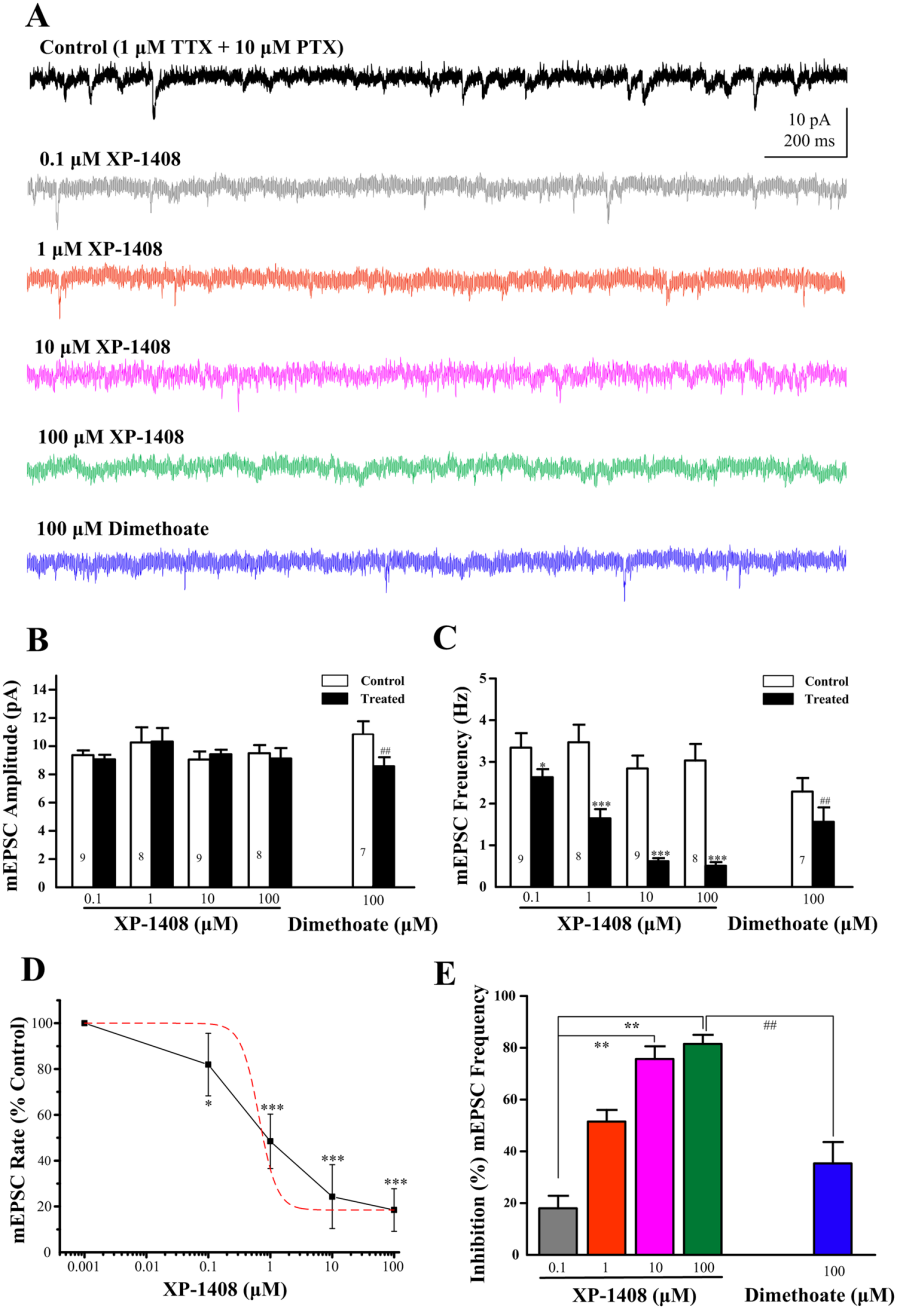
Effects of XP-1408 on cholinergic excitatory synaptic input to PNs. (**A**) Representative recordings show the effects of XP-1408 (0.1–100 μM) and 100 μM dimethoate on mEPSCs of PNs. (**B**) The mean mEPSCs amplitude in different concentrations of XP-1408 has no obvious change, but significantly decreased in 100 μM dimethoate. (**C**) The mean frequency of mEPSCs in control saline is significantly reduced by acute application of different concentrations of XP-1408 and 100 μM of dimethoate (*P < 0.05, **P < 0.01, ***P < 0.001 compared to control). Each bar indicates the mean ± SEM from the indicated number of neurons (n). (**D**) Concentration-response curve for XP-1408 inhibition of average mEPSC rate. Each symbol represents the mean ± SEM (n = 8–9). The IC_50_ value was 0.66 μM, determined from a dose-response logistic model (dashed line). (**E**) Plots of the percentage inhibition of mEPSC frequency 10 min after XP-1408 (0.1–100 μM) and dimethoate (100 μM) application, normalized to control values (100%). XP-1408 (0.1 μM) vs. XP-1408(10 μM), **P < 0.01; XP-1408(0.1 μM) vs. XP-1408(100 μM), **P < 0.01; XP-1408 (100 μM) vs. dimethoate (100 μM), ^##^ *P* < 0.01.

There is considerable evidence to assume that some OP insecticides can interact with multiple targets in the central and/or peripheral nervous systems in addition to AChE inhibition^2, 8^. Therefore we further investigated whether XP-1408 reproduced the actions of several OP insecticides that impact the cholinergic neurotransmission. In *Drosophila* CNS, fast cholinergic spontaneous postsynaptic currents have both an AP-dependent and an AP-independent component. As shown in Fig. 4A, XP-1408 could significantly inhibit the spontaneous activities in the presence of TTX, indicating that XP-1408 alters AP-dependent and/or AP-independent transmission at cholinergic synapse. To determine whether there were changes in synaptic transmission, we examined AP-independent transmitter release by recording miniature excitatory postsynaptic currents (mEPSCs). Due to the solubility, we added XP-1408 into the recording solution with a pipette. In the recording system, it took 2~3 minutes to reach equilibrium after compounds treatment. Thus, the data recorded after a 5-min bath-application were used for the analysis. The shorter latency of depolarization onset and the decreased input resistance induced by phoxim made the current baseline oscillate dramatically during the recording period. Considering the oscillation of membrane current in the presence of phoxim was not conducive to analyse mEPSCs, dimethoate, a widely used OP insecticide, was chosen as a control drug. Compared to control values, a 5 min bath-application of 100 μM of XP-1408 did not affect mEPSC amplitude (control, 9.51 ± 0.58pA vs. XP-1408, 9.13 ± 0.74pA; n = 8, *P* > 0.05, Fig. 5A and B), but it caused a significant decrease in frequency of mEPSCs (control, 3.04 ± 0.4 Hz vs. XP-1408, 0.51 ± 0.09 Hz; n = 8, ***P < 0.001, Fig. 5A and C). XP-1408 inhibited mEPSCs rate in a concentration-dependent manner with IC_50_ value of 0.66 μM (Fig. 5D). The inhibition ratio of mEPSCs frequency significantly differed from low concentration (0.1 μM) to higher concentration (10 μM and 100 μM) (10 μM, **P < 0.01; 100 μM, **P < 0.01). Noticeably, 100 μM of dimethoate decreased both amplitude and frequency of mEPSCs from 10.83 ± 0.94 pA to 8.58 ± 0.63 pA (**P < 0.01, n = 7) and 2.29 ± 0.32 Hz to 1.56 ± 0.35 Hz (**P < 0.01, n = 7), respectively (Fig. 5B and C). As with the effects of these two compounds on mEPSCs frequency, dimethoate was less potent than XP-1408 (^##^ *P* < 0.01, Fig. 5E).

The strength of synaptic transmission can be altered through modulation of transmitter release probability regulated by the Ca^2+^ signals in the presynaptic terminal, and postsynaptic responsiveness influenced by the number of postsynaptic receptors available and vesicle’s contents^25^. Our results showed that XP-1408 inhibited the frequency of mEPSCs in a concentration-dependent manner. By contrast, high concentration (100 μM) of dimethoate decreased both amplitude and frequency of mEPSCs, which was consisted with previous investigations in miniature end-plate currents^26^. The action mechanism of dimethoate was considered to be the direct action of itself by occupying the competitive ACh binding site or other non-competitive domains of nAChR, and the indirect persistent activation of nAChR by accumulating ACh following AChE inhibition^5, 27^. Both actions of dimethoate caused the blockage of neuromuscular or synaptic transmission and the persistent AChR desensitization, leading to paralysis of muscles and the death of insects. As mentioned before, XP-1408 had inhibitory effect on the frequency but not the amplitude of mEPSCs. This strongly suggested that XP-1408 was acting on presynaptic terminals to decrease the probability of spontaneous ACh vesicular release, without impacting the sensitivity of nAChRs in the postsynaptic area^28^. It has been demonstrated that any means increasing ACh concentration in the synaptic cleft could bring about the direct activation of presynaptic muscarinic ACh receptors (mAChRs). Because mAChRs is involved in the presynaptic negative feedback mechanism^8, 29^. The inhibitory effect of XP-1408 on cholinergic synaptic transmission may come from the feedback inhibition of ACh release and presynaptic calcium channel numbers and/or function. To conclude, the action sites and modes of XP-1408 on cholinergic synapses are different from that of dimethoate.

### Effects of XP-1408 on the voltage-dependent K^+^ currents

The RMP sets the driving force for synaptic currents and controls the activation of voltage-gated conductance. The opening of AChRs could increase the influx of the Na^+^ ions which caused a depolarization of postsynaptic membrane. However, this possibility was unlikelihood due to the presynaptic action site of XP-1408 in cholinergic synapses. In addition, the inability to block membrane depolarization by the pretreatment with TTX impelled K _v_ channels to be first considered as a likely cause. According to the gating, kinetic, and pharmacological properties, K _v_ currents in *Drosophila* central neurons are classified into 4-aminopyridine (4-AP)-sensitive, transient, inactivating I_K(A)_ and delayed-rectifier, sustained, non-inactivating I_K(DR)_^30, 31^. As two major classes of K _v_ currents in central neurons, I_K(A)_ channels played crucial roles in regulating neuronal excitability by modulating action potential firing frequency and slowing the rate of depolarization, whereas I_K(DR)_ channels function to promote the onset of repolarization and maintain the baseline membrane potential. In pharmacology, it has been found AChE inhibitors could affect central outward K^+^ channels^32^, such as tacrine, donepezil, which inhibited I_K(A)_ in rat hippocampal neurons^33^ and/or I_K(DR)_ in the larval muscles of *Drosophila* ^34^. Ca^2+^-dependent K^+^ currents also had been reported to underlie the mechanism of hyperexcitability induced by paraoxon in snail neurons^8^. However, in toxicology, there were rare studies about the relationship between AChE inhibitors such as OP insecticides with I_K(A)_ and I_K(DR_ channels^35–37^.

**Figure 6:**
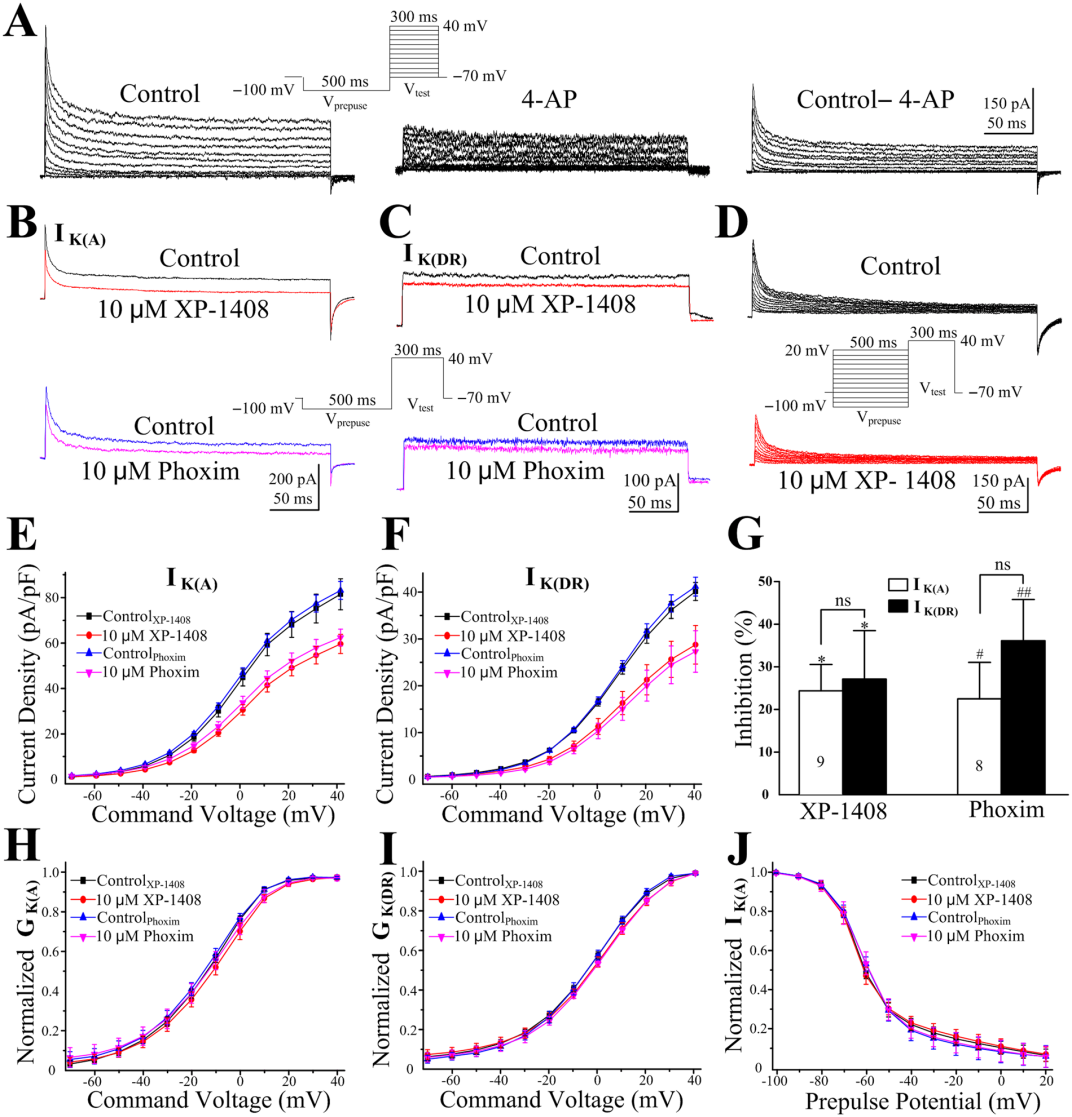
Effects of XP-1408 on I_K(A)_ and I_K(DR)_ in *Drosophila* PNs. (**A**) Representative voltage-gated potassium currents in PNs. Left: total outward potassium current stimulated with 300 ms depolarizing pulses from −70 to +40 mV in 10 mV steps following a hyperpolarizing prepulse of 500 ms to −100 mV (inset). Middle: I_K(DR)_ generated using the same protocol in the presence of 3 mM of 4-AP and 1 μM of TTX. Right: I_K(A)_ obtained by subtracting I_K(DR)_ from total. The peak of the subtracted currents was referred to as I_K(A)_. (**B**) Representative superimposed current traces of I_K(A)_ obtained at +40 mV in control conditions, 10 min after the application of 10 μM of XP-1408 and 10 μM of phoxim. (**C**) Representative superimposed current traces of I_K(DR)_ obtained at +40 mV in control conditions, 10 min after the addition of 10 μM of XP-1408 and 10 μM of phoxim. Inset is the pulse protocol. (**D**) Representative inactivation traces of I_K(A)_ before and after the addition of 10 μM of XP-1408. Inset is the pulse protocol. (**E**) The effects of 10 μM of XP-1408 and10 μM of phoxim on the I-V curves of I_K(A)_. (**F**) The effects of 10 μM of 1408 and10 μM of phoxim on the I-Vcurves of I_K(DR)_. (**G**) Percentage inhibition of I_K(A)_ and I_K(DR)_ 10 min after XP-1408 (10 μM) and phoxim (10 μM) application, normalized to control values (100%). ^(#)^*P < 0.05 vs. control (I_K(A)_ or I_K(DR)_), ^(##)^**P < 0.01 vs. control (I_K(A)_ or I_K(DR)_). ns, not significant, *P* > 0.05. Bar represent means ± SEM from indicated number of neurons. (**H**) The effects of 10 μM of XP-1408 and 10 μM of phoxim on the steady-state activation curves of I_K(A)_. (**I**)The effects of 10 μM of XP-1408 and 10 μM of phoxim on the steady-state activation curves of I_K(DR)_. (**J**) The effects of 10 μM of XP-1408 and 10 μM of phoxim on the steady-state inactivation curves of I_K(A)_.

To isolate I_K(DR)_ in PNs, the cells were bathed with Ca^2+^-free solution containing 1 μM TTX, 3 mM 4-AP to substantially block non-I_K(DR)_ currents. The neurons were held at −70 mV and then stepped to −100 mV for 500 ms (conditioning prepulse potential). Isolated outward currents were elicited by 300 ms depolarizing steps from −70 mV to +40 mV in increments of 10 mV (Fig. 6A). I_K(A)_ cannot be recorded in isolation from I_K(DR)_ because there are no selective blockers of insect I _K(DR)_. Therefore the I_K(A)_ was isolated by subtracting the current in the presence of 3 mM 4-AP from the total control current, yielding the 4-AP-sensitive I_K(A)_ (Fig. 6A). Based on the depolarization amplitude induced by XP-1408, 10 μM was chosen to record ionic currents. A typical example of XP-1408 (10 μM) on the density of I_K(A)_ and I _K(DR)_ was shown in Fig. 6B and C. Figure 6E and F summarized the current-voltage (I-V) relationships of I_K(A)_ and I_K(DR)_ after XP-1408 and phoxim administration, respectively. At a +40 mV step pulse, 10 μM of XP-1408 application inhibited 24.2 ± 6.2% (*P < 0.05 vs. control, n = 9) of the baseline I_K(A)_, and 27.1 ± 11.4% (*P < 0.05 vs. control, n = 9) of the baseline I_K(DR)_. But the inhibition rate of I_K(A)_ was not different from that of I_K(DR)_. Similarly, at a concentration of 10 μM, phoxim was also able to significantly reduce the peak I_K(A)_ amplitude by 22.5 ± 8.6% (^#^ *P* < 0.05 vs. control, n = 8) and the I_K(DR)_ amplitude by 36.2 ± 9.6% (^##^ *P* < 0.01vs. control, n = 8) at +40 mV depolarizing pulse. No significant differences were found in the inhibition percentage of I_K(A)_ and I_K(DR)_ after phoxim administration. In addition, there were no significant differences between XP-1408 and phoxim on the magnitude of I_K(A)_ and I_K(DR)_, respectively (Fig. 6G).

Further, voltage dependencies of activation and inactivation were compared between control and in presence of XP-1408 and phoxim to determine whether changes in toxin administration could contribute to the reduced current density. In presence of XP-1408 (10 μM), the voltage dependence and sensitivity of activation for I_K(A)_ and I_K(DR)_ in PNs were both significantly changed, as measured by comparing the peak conductance (G). G was then normalized with respect to the calculated maximal conductance G_max_. The half activation potential (V_1/2_) and slope factor (*k*) in the I_K(A)_ activation curve were −13.3 ± 0.4 mV and 12.2 ± 0.2 under control conditions, −10.6 ± 0.7 mV (*P < 0.05 vs. control, n = 9) and 13.1 ± 0.3 (*P < 0.05 vs. control, n = 9) after XP-1408 application, respectively. Mean values for V_1/2_ and *k* in the activation of I_K(DR)_ were −1.5 ± 0.4 mV and 13.9 ± 0.1 under control conditions, 1.5 ± 0.7 mV (*P < 0.05 vs. control, n = 9) and 14.6 ± 0.3 (*P < 0.05 vs. control, n = 9) after XP-1408 application, respectively. However, while the V_1/2_ for I_K(A)_ and I _K(DR)_ was significantly shifted in the presence of 10 μM phoxim (I_K(A)_: control = −13.9 ± 0.4 mV vs. phoxim = −11.7 ± 0.9 mV, ^#^ *P* < 0.05, n = 8; I_K(DR)_: control = −1.9 ± 0.5 mV vs. phoxim = 1.7 ± 0.4 mV, ^##^ *P* < 0.01, n = 8), the slope factors remained unchanged for I_K(A)_, but increased for I _K(DR)_ (control: 13.6 ± 0.1 vs. phoxim: 14.5 ± 0.3, ^##^ *P* < 0.01) (Fig. 6H and I; Table 3).

**Table 3:** Changes in kinetics of I_K(A)_ and I_K(DR)_ activation/inactivation properties in response to XP-1408 and phoxim applications.

	I_K(A)_				I_K(DR)_	
	V_1/2act_(mV)	k_act_	V_1/2inact_ (mV)	k_inact_	V_1/2act_ (mV)	k_act_
Control XP-1408	−13.3 ± 0.4	12.2 ± 0.2	−61.7 ± 0.3	8.9 ± 0.1	−1.5 ± 0.4	13.9 ± 0.1
10 μM XP-1408	−10.6 ± 0.7*	13.1 ± 0.3*	−62.5 ± 0.5	9.3 ± 0.3	1.5 ± 0.7*	14.6 ± 0.3*
Control Phoxim	−13.9 ± 0.4	12.2 ± 0.2	−60.3 ± 0.3	8.8 ± 0.1	−1.9 ± 0.5	13.6 ± 0.1
10 μM Phoxim	−11.7 ± 0.9^#^	12.8 ± 0.2	−60.5 ± 0.9	9.0 ± 0.2	1.7 ± 0.4^# #^	14.5 ± 0.3^# #^

Values are the mean ± SEM, *P < 0.05 vs. control_XP-1408_, ^#^ *P* < 0.05 vs. control_Phoxim_, ^# #^ *P* < 0.01 vs. control_Phoxim_.

The inactivation characteristics of I_K(A)_ were examined using a two-pulse voltage protocol, in which the K^+^ currents were activated by 300 ms test pulses to +40 mV after 500 ms conditioning prepulses ranging from −100 to +20 mV in 10 mV increments (Fig. 6D). The inactivation curve was plotted as the normalized peak current of I_K(A)_ against the potential of the conditioning prepulse (Fig. 6J). Both XP-1408 and phoxim had no effects on inactivation of I_K(A)_ (Fig. 6J; Table 2).

To sum up, our study showed that XP-1408 (10 μM) remarkably reduced the amplitudes of I_K(A)_ and I_K(DR)_, and also significantly affected the activation curves of them in a depolarizing direction without any influence on the inactivation curves of I_K(A)_. These implied that the depression of I_K(A)_ and I_K(DR)_ by XP-1408 was in a voltage-dependent manner, mainly due to deferred activation of both currents by changing the intrinsic activation-gating properties. Moreover, the positive shift in the activation of I_K(DR)_ (about 3.0 mV) was slightly more than that of I_K(A)_ (about 2.8 mV). It indicated that XP-1408 exhibited comparable blocking effects on these two kinds of currents, however, the blocking potency on I_K(DR)_ was slightly stronger than that of I_K(A)_. The effects of phoxim on I_K(A)_ and I_K(DR)_ were similar to that of XP-1408, suggesting these two compounds may act in a similar fashion. Thus, we speculated that the blockage of I_K(A)_ may contribute to the increasing spontaneous activities in the early stage after XP-1408 administration, whereas the inhibition of I_K(DR)_ most likely account for membrane depolarization.

### Effects of XP-1408 on the voltage-dependent Na^+^ currents

**Figure 7:**
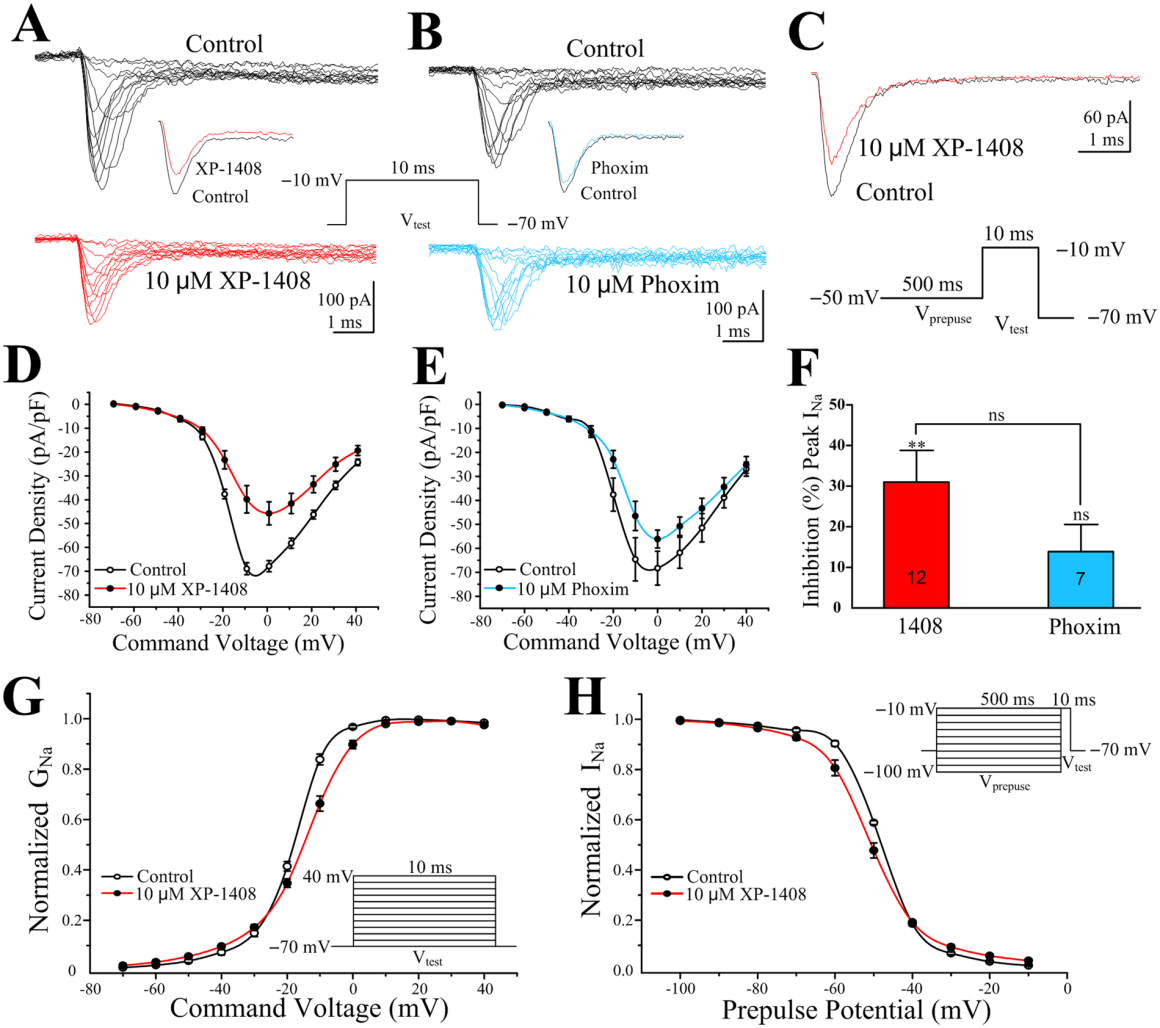
Effects of XP-1408 on whole-cell Na_V_ channel currents in *Drosophila* PNs. (**A**) Typical families of I_Na_ recorded in control conditions and 10 min after the application of 10 μM of XP-1408. Inset is the high time resolution of I_Na_ obtained at −10 mV. (**B**) Typical families of I_Na_ recorded in control conditions and 10 min after the application of 10 μM of phoxim. Inset is the high time resolution of I_Na_ obtained at −10 mV. (**C**) Representative superimposed inactivation traces of I_Na_ obtained at prepulse potential of −10 mV in control conditions, and 10 min after the application of 10 μM of XP-1408 (upperpanel). Pulse protocol is shown in the bottom panel. (**D**) The effects of 10 μM of XP-1408 on the I-V curves of I_Na_. (**E**) The effects of 10 μM of phoxim on the I-V curves of I_Na_. (**F**) Percentage inhibition of I_Na_ 10 min after XP-1408 (10 μM) and phoxim (10 μM) application, normalized to control values (100%). **P < 0.01 vs. control (I_Na_). ns, not significant, *P* > 0.05. Bar represent means ± SEM from indicated number of neurons. (**G**) The effects of 10 μM of XP-1408 on the steady-state activation curve of I_Na_. All data are expressed as the mean ± SEM of 12 cells. (**H**) The effects of 10 μM of XP-1408 on the steady-state inactivation curve of I_Na_. All data are expressed as the mean ± SEM of 12 cells.

Based on the molecular docking results, we further investigated the effect of XP-1408 on insect Na_v_ channels (I_Na_). The I_Na_ played a major role in neuronal excitability by regulating action potential initiation and propagation, making them the most commonly targeted ion channel family by toxins including insecticides, drugs and neurotoxins^29^. Whole-cell TTX-sensitive I_Na_ was recorded from PNs in the voltage-clamp configuration by depolarizing −10 mV voltage steps from −70 to +40 mV from a holding potential of −70 mV (Fig. 7A and B). After 10 min application, 10 μM XP-1408 obviously depressed the I-V curves of I_Na_ (Fig. 7D) and I_Na_ peak current densities was deceased by 31.1 ± 7.8% (**P < 0.01 vs. control, n = 12; Fig. 7F). The effects of phoxim (10 μM) on I_Na_ were also examined. As shown in Fig. 7E and F, neither the I-V curves of I_Na_ nor the I_Na_ peak current densities were significantly inhibited after phoxim application.

The voltage dependence of activation and inactivation of I_Na_ were also investigated to determine if changes in these properties could contribute to the decrease in current density induced by XP-1408. Activation curves were generated using a series of 10-ms depolarizing voltage steps from −70 mV to +40 mV in 10 mV increments. In the presence of 10 μM of XP-1408, a positive shift in half-activation voltage by 3.6 mV was observed (from V_1/2_ = −18.1 ± 0.5 mV in control to −14.5 ± 0.7 mV in the presence of XP-1408 (**P < 0.01, n = 12). Meanwhile, the slope factors were also altered significantly (control: 5.4 ± 0.1 vs. XP-1408: 7.6 ± 0.3, **P < 0.01) (Fig. 7G). But phoxim (10 μM) had no effect on activation parameters. Steady-state inactivation of I_Na_ was measured from a holding potential of −70 mV; a series of 500 ms conditioning prepulses were delivered in 10 mV increments from −100 to −10 mV, followed by a 10-ms test pulse to 0 mV. An example inactivation was demonstrated with traces of I_Na_ before and 10 min following the addition of 10 μM of XP-1408 at prepulse potential of −50 mV as shown in Fig. 7C. Steady-state inactivation curves were obtained by plotting the amplitude of the peak inward current measured during test pulse against the conditioning potential. Phoxim (10 μM) had no effects on inactivation parameters, too. In contrast, XP-1408 (10 μM) caused a small, but significant negative shift in the inactivation curve (control: −48.2 ± 0.2 vs. XP-1408: −51.0 ± 0.8, *P < 0.05, n = 12), and increased the slope value (control: 5.1 ± 0.1 vs. XP-1408: 6.2 ± 0.2, *P < 0.05) (Fig. 7H).

In summary, in the later stage after the application of XP-1408, the spontaneous activities gradually reduced and eventually disappeared, which implied that Na_v_ may also be the target of XP-1408. Indeed, in the presense of XP-1408, the peak current density of I_Na_ was obviously decreased due to the alterations in voltage dependence and sensitivity of both activation and inactivation. The positive shift in the activation curve and negative shift in the steady-state inactivation curve, suggested that XP-1408 blocked the Na_v_ channels in a state-dependent manner and could be characterized as a gating-modifier. However, phoxim had no significant inhibition either on the peak current of I_Na_ or its kinetic properties, which was consistent with the molecular docking results. Thus, it indicated that the mode of action on Na_v_ channels distinguished XP-1408 from typical OP insecticides.

The above results suggested that both K _v_ and Na_v_ channel were the targets of XP-1408, and the altered neuronal excitability in PNs was associated with the simultaneous blocking of these two types of channel. These may contribute to the underlying mechanisms through which XP-1408 affected the development of *Drosophila* larvae. Further radioligand binding studies and electrophysiological experiments were required to determine the precise binding sites of XP-1408 on insect K _v_ and Na_v_ channels, the effects on other types of potassium channels.

### Effect of XP-1408 on development of *Drosophila* larvae

Neurotransmitters such as ACh played unique trophic roles in organism development by disrupting the timing or intensity of neurotrophic actions through receptor mechanisms and cell signaling cascades^38^. Accordingly, many drugs and toxins that promoted or interfered with neurotransmitter function could evoke functional changes of nervous and other system. Indeed, it has reported that OP insecticides could block signal transduction pathways, inhibit DNA and RNA synthesis, and induce oxidative stress, leading to apoptosis and necrosis^39, 40^. Additionally, apoptosis and cell differentiation also required the mediation of ion channels, such as K^+^, Ca^2+^ and Cl^-^ channels^41, 42^. In our experiments, XP-1408 could induce robust developmental lag in the larva-to-pupa transition (Fig. 2A and B). However, to date, these phenomena on insects have not been observed in the typical OP insecticides. Thus, it could be speculated that XP-1408 may possess additional targets besides of AChE, which was consistent with our electrophysiology results. The reasons of delayed development may be attributed to the following points: 1) brain development was slowed by blocking cholinergic neurotransmission; 2) the suppression of neural cells proliferation and initiation of apoptosis were triggered through blocking membrane K _v_ and/or Na_v_ channels^42, 43^; 3) similar to classical OP insecticides, XP-1408 may disturb the growth, reproduction, and oxidative stress responses by reducing food consumption and digestion rate and inducing the production of reactive oxygen in gland and nervous system^44^; and 4) the synergistic actions of above factors.

## Conclusion

In this study, novel phosphorus-containing arylisoxazoline derivatives were synthesized, and their biological activities were also studied. The aryisoxazoline-phosphorothioate conjugate named XP-1408 displayed potential novel growth inhibition activity. The possible physiological targets were investigated by using molecular docking and whole-cell patch clamp recording. The multiple effects that involve inhibitoion of AChE, suppression of cholinergic synaptic transmission, and attenuation of K _v_ and Na_v_ channels, may contribute to the underlying mechanisms of the delayed development of *Drosophila* larvae, and prompt XP-1408 as a lead compound of OP insecticides with a new mode of action. We believe that this study will provide a basis for further research on formulating novel OP insecticides. Detailed studies focusing on the substitution pattern and the isolation of the enantiomer are under way.

## Materials and Methods

### Instruments and chemicals

Melting points were measured on a YRT-3 melting point apparatus and were uncorrected. NMR spectra were recorded at 22 °C on a Bruker AV 600 MHz spectrometer, and chemical shifts (δ) are given in parts per million (ppm). The ^1^H and ^13^C chemical shift were referenced to the CDCl_3_ solvent peaks at δ_H_ 7.26 and δ_C_ 77.0 ppm, respectively. Electron ionization mass spectra (EI-MS) were determined by Waters micromass ZQ-2000 mass spectrometer, and HRMS were acquired on an Angilent 1290–6540B UPLC-Q-TOF mass spectrometer, respectively. Flash column chromatography was performed with silica gel (200–300 mesh) purchased from Qingdao Haiyang Chemical Co. Ltd. All chemical reagents were obtained from Aldrich Chemical Co. and treated with standard methods before use.

### Synthetic procedures

The target compounds I were synthesized using *p*-hydroxybenzaldehyde **1** as the starting material (see Fig. 1). The experimental procedures are as following: firstly, *p*-hydroxybenzaldoxime **2** was synthesized from 10 mmol of the aldehyde 1, 30 mmol of NH_2_OH·HCl and 30 mmol of NaOH dissolved in 100 mL of water at room temperature. In the next step, 5 mmol of the resultant aldoxime **2** reacted with 12.5 mmol of aryl ethene, 5 mmol of KCl and 7.5 mmol of oxone in 50 mL of water in order to obtain 3-(*p*-hydroxyphenyl)−5-phenyl isoxazoline **3** ^45^. Then, to a stirring solution of 15 mL of dry dioxane, 2 mmol of the aforementioned isoxazoline **3**, 6 mmol of *O*, *O*-dialkyl thiophosphoryl chloride and 6 mmol of NEt_3_ were added in turn under nitrogen atmosphere at 0 °C. The reaction was under stirring for approx. 12 h at 85 °C. The reaction was checked according to TLC analysis. The mixture was separated by flash column chromatography, using petroleum ether and ethyl acetate mixture (in proportions of 10: 1) as the eluent to give the target compounds I. The similar experimental protocol was performed to obtain compounds II, wherein the intermediates 7 were synthesized by using iron powder and glacial acetic acid under reflux (molar ratio of reactants n_6_: n_Fe_ = 1: 5)^46^.

#### Data for O, O-dimethyl O-(4-(5-phenyl-4, 5-dihydroisoxazol-3-yl) phenyl) phosphorothioate (**I-1**)

yellow oil; yield 53%; ^1^H NMR (600 MHz, CDCl_3_) δ 7.73–7.65 (m, 2H, Ph-H), 7.40–7.35 (m, 4H, Ph-H), 7.34–7.30 (m, 3H, Ph-H), 5.74 (dd, *J* = 11.0, 8.2 Hz, 1H, 2-isoxazline-CH), 3.85 (dd, *J* = 13.1, 0.7 Hz, 6H, OCH_3_), 3.76 (dd, *J* = 16.6, 11.0 Hz, 1H, 2-isoxazline-CH_2_), 3.32 (dd, *J* = 16.6, 8.2 Hz, 1H, 2-isoxazline-CH_2_); ^13^C NMR (150 MHz, Chloroform-*d*) δ 155.14 (2-isoxazline-NC), 151.64, 140.71, 128.78, 128.34, 128.29, 127.04, 125.82, 121.20, 82.76 (2-isoxazline-CH), 53.85 (d, ^2^ *J*_P,C_ = 6.5 Hz, OCH_3_), 43.10 (2-isoxazline-CH_2_); ^31^P NMR (243 MHz, Chloroform-*d*) δ 64.81. MS (EI): m/z calcd for C_17_H_18_NO_4_PS [M+2H]^+^ 365.4, found 365.2.

#### Data for O, O-diethyl O-(4-(5-phenyl-4, 5-dihydroisoxazol-3-yl) phenyl) phosphorothioate (**I-2/XP-1408**)

yellow oil; yield 66%; ^1^H NMR (600 MHz, Chloroform-*d*) δ 7.70–7.66 (m, 2H, Ph-H), 7.40–7.36 (m, 4H, Ph-H), 7.34–7.30 (m, 1H, Ph-H), 7.25–7.21 (m, 2H, Ph-H), 5.74 (dd, *J* = 11.0, 8.2 Hz, 1H, 2-isoxazline-CH), 4.29–4.20 (m, 4H, OC*H*_2_CH_3_), 3.76 (dd, *J* = 16.6, 11.0 Hz, 1H, 2-isoxazline-CH_2_), 3.32 (dd, *J* = 16.6, 8.2 Hz, 1H, 2-isoxazline-CH_2_), 1.37 (td, *J* = 7.1, 0.8 Hz, 6H, OCH_2_C*H*_3_); ^13^C NMR (150 MHz, Chloroform-*d*) δ 155.26 (2-isoxazline-NC), 152.05, 140.80, 128.78, 128.27, 128.09, 126.58, 125.84, 121.39, 82.69 (2-isoxazline-CH), 65.24 (d, ^2^ *J*_P,C_ = 5.7 Hz, O*C*H_2_CH_3_), 43.19 (2-isoxazline-CH_2_), 15.92 (d, ^3^ *J*_P,C_ = 7.4 Hz, OCH_2_ *C*H_3_); ^31^P NMR (243 MHz, Chloroform-*d*): δ 62.77. HRMS (ESI): m/z calcd for C_19_H_22_NO_4_PS [M+H]^+^ 392.1007, found 392.1080.

#### Data for O-(4-(5-(4-(tert-butyl)phenyl)−4, 5-dihydroisoxazol-3-yl) phenyl) O, O-dimethyl phosphorothioate (**I-3**)

yellow oil; yield 37%; ^1^H NMR (600 MHz, Chloroform-*d*) δ 7.72–7.67 (m, 2H, Ph-H), 7.41–7.39 (m, 2H, Ph-H), 7.33–7.30 (m, 4H, Ph-H), 5.73 (dd, *J* = 11.0, 8.3 Hz, 1H, 2-isoxazline-CH), 3.81 (dd, *J* = 13.2, 0.7 Hz, 6H, OCH_3_), 3.73 (dd, *J* = 16.6, 11.0 Hz, 1H, 2-isoxazline-CH_2_), 3.34 (dd, *J* = 16.6, 8.3 Hz, 1H, 2-isoxazline-CH_2_), 1.32 (s, 9H, C(CH_3_)_3_); ^13^C NMR (150 MHz, Chloroform-*d*): δ 155.83 (2-isoxazline-NC), 151.28, 148.24, 137.83, 129.92, 128.50, 125.71, 125.65, 122.07, 82.23 (2-isoxazline-CH), 53.42 (d, ^2^ *J*_P,C_ = 6.2 Hz, OCH_3_), 43.21 (2-isoxazline-CH_2_), 34.59 (*C*(CH_3_)_3_), 31.31 (C(*C*H_3_)_3_); ^31^P NMR (243 MHz, Chloroform-*d*): δ 64.58. MS (EI): m/z calcd for C_21_H_26_NO_4_PS [M+2H]^+^ 421.5, found 421.6.

#### Data for O-(4-(5-(4-(tert-butyl) phenyl)-4, 5-dihydroisoxazol-3-yl) phenyl) O, O-diethyl phosphorothioate (**I-4**)

yellow oil; yield 45%; ^1^H NMR (600 MHz, Chloroform-*d*) δ 7.71–7.64 (m, 2H, Ph-H), 7.43–7.38 (m, 2H, Ph-H), 7.32–7.27 (m, 2H, Ph-H), 7.25–7.22 (m, 2H, Ph-H), 5.73 (dd, *J* = 11.0, 8.3 Hz, 1H, 2-isoxazline-CH), 4.33–4.28 (m, 4H, OC*H*_2_CH_3_), 3.70 (dd, *J* = 16.6, 11.0 Hz, 1H, 2-isoxazline-CH_2_), 3.31 (dd, *J* = 16.6, 8.3 Hz, 1H, 2-isoxazline-CH_2_), 1.40 (td, *J* = 7.1, 0.8 Hz, 6H, OCH_2_C*H*_3_), 1.32 (s, 9H, C(CH_3_)_3_); ^13^C NMR (150 MHz, Chloroform-*d*): δ 154.47 (2-isoxazline-NC), 151.49, 148.05, 137.37, 128.73, 125.93, 125.73, 125.63, 121.58, 82.91(2-isoxazline-CH), 65.50 (d, ^2^ *J*_P,C_ = 5.8 Hz, O*C*H_2_CH_3_), 42.64 (2-isoxazline-CH_2_), 34.61 (*C*(CH_3_)_3_), 31.29 (C(*C*H_3_)_3_), 15.91 (d, ^3^ *J*_P,C_ = 7.7 Hz, OCH_2_ *C*H_3_); ^31^P NMR (243 MHz, Chloroform-*d*): δ 62.47. MS (EI): m/z calcd for C_23_H_30_NO_4_PS [M+2H]^+^ 449.5, found 449.3.

#### Data for O, O-dimethyl (4-(5-phenyl-4, 5-dihydroisoxazol-3-yl) phenyl) phosphoramidothioate (**II-1**)

pale yellow solid; yield 56%; mp 68–70 °C; ^1^H NMR (600 MHz, Chloroform-*d*) δ 7.64–7.59 (m, 2H, Ph-H), 7.41–7.34 (m, 4H, Ph-H), 7.34–7.29 (m, 1H, Ph-H), 7.14–7.09 (m, 2H, Ph-H), 5.94 (d, *J* = 13.6 Hz, 1H, NH), 5.72 (dd, *J* = 11.0, 8.2 Hz, 1H, 2-isoxazline-CH), 3.87 (dd, *J* = 12.7, 0.6 Hz, 3H, OCH_3_), 3.75 (dd, *J* = 16.5, 11.0 Hz, 1H, 2-isoxazline-CH_2_), 3.61 (dd, *J* = 12.8, 0.6 Hz, 3H, OCH_3_), 3.31 (dd, *J* = 16.5, 8.2 Hz, 1H, 2-isoxazline-CH_2_); ^13^C NMR (150 MHz, Chloroform-*d*): δ 155.52 (2-isoxazline-NC), 140.97, 140.59, 128.75, 128.21, 128.03, 125.85, 123.68, 118.15, 82.42 (2-isoxazline-CH), 52.97 (d, ^2^ *J*_P,C_ = 6.3 Hz, OCH_3_), 43.28 (2-isoxazline-CH_2_); ^31^P NMR (243 MHz, Chloroform-*d*): δ 52.23. MS (EI): m/z calcd for C_17_H_19_N_2_O_3_PS [M+2H]^+^ 364.4, found 364.5.

#### Data for O, O-diethyl (4-(5-phenyl-4, 5-dihydroisoxazol-3-yl) phenyl) phosphoramidothioate (**II-2**)

pale yellow solid; yield 68%; mp 89–91 °C; ^1^H NMR (600 MHz, Chloroform-*d*) δ 7.61–7.58 (m, 2H, Ph-H), 7.40–7.33 (m, 4H, Ph-H), 7.32–7.29 (m, 1H, Ph-H), 7.13–7.07 (m, 2H, Ph-H), 6.12 (d, *J* = 13.8 Hz, 1H, NH), 5.72 (dd, *J* = 11.0, 8.2 Hz, 1H, 2-isoxazline-CH), 4.32–4.16 (m, 4H, OC*H*_2_CH_3_), 3.74 (dd, *J* = 16.6, 11.0 Hz, 1H, 2-isoxazline-CH_2_), 3.29 (dd, *J* = 16.6, 8.2 Hz, 1H, 2-isoxazline-CH_2_), 1.35 (t, *J* = 7.1 Hz, 3H, OCH_2_C*H*_3_), 1.31 (t, *J* = 7.1 Hz, 3H, OCH_2_C*H*_3_); ^13^C NMR (150 MHz, Chloroform-*d*) δ 155.61 (2-isoxazline-NC), 140.94, 140.65, 128.75, 128.21, 127.90, 125.88, 123.75, 118.51, 82.44 (2-isoxazline-CH), 65.74 (d, ^2^ *J*_P,C_ = 5.6 Hz, O*C*H_2_CH_3_), 65.51 (d, ^2^ *J*_P,C_ = 5.3 Hz, O*C*H_2_CH_3_), 43.24 (2-isoxazline-CH_2_), 15.78 (d, ^3^ *J*_P,C_ = 7.9 Hz, OCH_2_ *C*H_3_), 15.72 (d, ^3^ *J*_P,C_ = 8.4 Hz, OCH_2_ *C*H_3_); ^31^P NMR (243 MHz, Chloroform-*d*) δ 50.70. HRMS (ESI): m/z calcd for C_19_H_23_N_2_O_3_PS [M+H]^+^ 391.1167, found 391.1242.

#### Data for O, O-dimethyl (4-(5-(4-(tert-butyl) phenyl)-4, 5-dihydroisoxazol-3-yl) phenyl) phosphoramidothioate (**II-3**)

pale yellow solid; yield 42%; mp 91–92 °C; ^1^H NMR (600 MHz, Chloroform-*d*) δ 7.61–7.53 (m, 2H, Ph-H), 7.39–7.37 (m, 2H, Ph-H), 7.33–7.31 (m, 2H, Ph-H), 7.12–7.07 (m, 2H, Ph-H), 5.79 (d, *J* = 13.6 Hz, 1H, NH), 5.71 (dd, *J* = 11.0, 8.3 Hz, 1H, 2-isoxazline-CH), 3.83 (dd, = 12.7, 0.6 Hz, 3 H, OCH_3_), 3.71 (dd, *J* = 16.6, 11.0 Hz, 1H, 2-isoxazline-CH_2_), 3.59 (dd, *J* = 12.5, 0.6 Hz, 3H, OCH_3_), 3.33 (dd, *J* = 16.6 Hz, 8.3 Hz, 1H, 2-isoxazline-CH_2_), 1.33 (s, 9H, C(CH_3_)_3_); ^13^C NMR (150 MHz, Chloroform-*d*): δ 155.51 (2-isoxazline-NC), 151.31, 140.35, 137.76, 128.62, 127.75, 125.67, 124.01, 118.43, 82.40 (2-isoxazline-CH), 53.01 (d, ^2^ *J*_P,C_ = 6.5 Hz, OCH_3_), 43.39 (2-isoxazline-CH_2_); 34.60 (*C*(CH_3_)_3_), 31.32 (C(*C*H_3_)_3_); ^31^P NMR (243 MHz, Chloroform-*d*): δ 52.24. MS (EI): m/z calcd for C_21_H_37_N_2_O_3_PS [M+2H]^+^ 420.5, found 420.4.

#### Data for O, O-diethyl (4-(5-(4-(tert-butyl) phenyl)-4, 5-dihydroisoxazol-3-yl) phenyl) phosphoramidothioate (**II-4**)

pale yellow solid; yield 51%; mp 99–101 °C; ^1^H NMR (600 MHz, Chloroform-*d*) δ 7.64–7.57 (m, 2H, Ph-H), 7.42–7.37 (m, 2H, Ph-H), 7.35–7.29 (m, 2H, Ph-H), 7.10–7.05 (m, 2H, Ph-H), 5.88 (d, *J* = 13.8 Hz, 1H, NH), 5.70 (dd, *J* = 10.9, 8.3 Hz, 1H, 2-isoxazline-CH), 4.33–4.17 (m, 4H, OC*H*_2_CH_3_), 3.71 (dd, *J* = 16.5, 10.9 Hz, 1H, 2-isoxazline-CH_2_), 3.32 (dd, *J* = 16.5, 8.3 Hz, 1H, 2-isoxazline-CH_2_), 1.37 (td, *J* = 7.1 Hz, 0.6 Hz, 3H, OCH_2_C*H*_3_), 1.34–1.31 (m, 12H, OCH_2_C*H*_3_+C(CH_3_)_3_); ^13^C NMR (150 MHz, Chloroform-*d*) δ 155.55 (2-isoxazline-NC), 151.33, 140.47, 137.76, 128.56, 127.89, 125.68, 124.03, 118.49, 82.40 (2-isoxazline-CH), 65.79 (d, ^2^ *J*_P,C_ = 5.7 Hz, O*C*H_2_CH_3_), 65.49 (d, ^2^ *J*_P,C_ = 5.5 Hz, O*C*H_2_CH_3_), 42.97 (2-isoxazline-CH_2_), 34.60 (*C*(CH_3_)_3_), 31.31 (C(*C*H_3_)_3_), 15.77 (d, ^3^ *J*_P,C_ = 7.5 Hz, OCH_2_ *C*H_3_), 15.71 (d, ^3^ *J*_P,C_ = 7.6 Hz, OCH_2_ *C*H_3_); ^31^P NMR (243 MHz, Chloroform-*d*) δ 50.83. MS (EI): m/z calcd for C_23_H_31_N_2_O_3_PS [M+2H]^+^ 448.5, found 448.3.

### Bioassay

The insecticidal activity of the target compounds against 2nd-instar *S*. *litura* larvae was evaluated by using leaf-dip method at a concentration of 100 mg/L^47^. The *S*. *litura* larvae were provided by the key laboratory of natural pesticide and chemical biology of the Ministry of Education, South China Agricultural University, P. R. China. All the final compounds were dissolved in 1 mL of DMSO. The commercial OP insecticides dimethoate and phoxim were chosen as positive control against the above test insect under the same conditions. Meanwhile, DMSO in sterile DDW (double-distilled water) was served as a blank control. The cassava leaves were cut and dipped into the test solution for 3s with agitation, set in the shade to dry, and then placed in a tube lined with a piece of cotton gauze. Five replicates were performed at 25 °C, and each replicate contained 20 test insects. Percentage mortalities were examined 72 h after treatment.

Meanwhile, in order to evaluate the growth inhibition activity of XP-1408, a study on developmental timing measurement of *D. melanogaster* (wild-type Canton-S) was carried out. The flies were cultured on standard food at 25 °C under a 12 h light/dark photoperiod according to our previous report^48^. The insect development protocol was referred to our former procedures^49^. Firstly, adult flies were given 1 h to lay any retained eggs in fresh plates. Then they were transferred to fresh plates to oviposit for 2 h. At 72 h AEL, synchronized larvae (third-instar) were transferred to fresh food with 25, 50, 100 mg/L of XP-1408 dissolved in DMSO (n = 20 larvae per tube), respectively. Fresh food containing DMSO was served as a blank control. Four independent populations were assayed for each treatment. Pupae were surveyed in 12 h intervals. Time “0” was defined as 2 h after the initiatory oviposition. The mean time of pupariation was calculated according to the pupal survey.

### Molecular docking

The molecular docking studies were performed using the Leadit 2.1.8 program to investigate the interaction between the compound and sodium channel (PDB ID: 4EKW), as well as the interaction between the compound and acetylcholinesterase (PDB ID: 1DX4). The water molecules and the ligand were deleted. Polarhydrogen was added and the Gasteiger charge was applied. Other parameters were default settings. The structure of XP-1408 was sketched by Gviewer. And the full geometry optimizations were performed with the Gaussian09 program package using the hybrid density functional theory (B3LYP) method and 6–31 G (D) basis set. All calculations were carried out on a Dell T7500 server with dual XEON 5660 cores, and a RedHat linux operating system. Visualization of the docked pose has been done by using PyMol molecular graphics program.

### Electrophysiological recordings

All experiments were performed on *D. melanogaster* (Canton-S, as mentioned above) flies 2 days before eclosion. The preparation of flies whole brains was based on an approach described previously^50^. After isolation, the brain was transferred instantly to the perfusion chamber containing the normal recording solution (101 mM NaCl, 1 mM CaCl_2_, 4 mM MgCl_2_, 3 mM KCl, 5 mM glucose, 1.25 mM NaH_2_PO_4_, 20.7 mM NaHCO_3_, pH = 7.2, 250 Osm) with the anterior face of the brain up.

Whole cell voltage- and current-clamp configurations were used to measure current and voltage changes in PNs. Patch pipettes were pulled from borosilicate glass with resistances of 9–12 MΩ filled with normal internal solution (102 mM K-gluconate, 0.085 mM CaCl_2_, 1.7 mM MgCl_2_, 17 mM NaCl, 0.94 mM EGTA, 8.5 mM HEPES, pH = 7.2, 235 Osm). Normal external and internal solutions were used to measure cholinergic mEPSCs with addition of TTX (1 μM) voltage-gated sodium blocker, and picrotoxin (PTX, 10 μM) GABA receptor antagonist in the external solutions. For measurements of Na^+^ current, CaCl_2_ was omitted, tetraethylammonium chloride (TEA, 15 mM) and 4-AP (3 mM) were added to the normal external solution. The pipette solution contained (mM): 102 D-gluconic acid, 102 CsOH, 0.085 CaCl_2_, 1.7 MgCl_2_, 17 NaCl, 0.94 EGTA, 8.5 HEPES and 4 ATP, pH 7.2, 235 Osm. For recordings of K^+^ current, TTX (1 μM) and 4-AP (3 mM) were added to the normal external solution with the removal of CaCl_2_. Pipette solution was the same as standard internal solution. Signals were acquired by an Axon-700B multi-clamp amplifier (Axon Instruments, Foster City, CA), digitized at 5~20 kHz with low-pass filtered at 2 kHz. RMP were measured under zero current-clamp condition immediately after breaking into the cell. All recordings were made at room temperature, and only a single neuron was examined in each brain.

### Curve-fitting and statistical analysis

Data analyses were completed by the pClamp 10 Clamp fit software (Molecular Devices), Sigma Stat (Systat Software, San Jose, CA), Mini analysis software (Synaptosoft, Decatur, GA, USA), and Origin 8.6 software (OriginLab, Northampton, MA). All data are presented as mean ± SEM. Statistical significance was assessed using a paired or unpaired Student’s t test and ANOVA with Bonferroni post hoc test.

To measure steady-state activation, incrementing voltage pulses were applied from a constant holding potential (for details see Results and figure legends). The voltage dependences of voltage-dependent K^+^ and Na^+^ currents were determined by converting the peak currents to peak conductances (G). Normalized conductance (G/Gmax) was plotted against test pulse voltage and the data were fitted using a Boltzmann equation of the form: G/G_max_ = 1/(1 + exp[(V_m_ − V_1/2_)/k)], in which V_m_ is the membrane potential, V_1/2_ is the voltage of half-maximal activation, and k is the slope factor. Steady-state inactivation of voltage-dependent currents was measured from a constant holding potential. Incrementing prepulses were followed by a constant test pulse, for which the peak currents were measured. Normalized peak current (I/I_max_) was plotted against the prepulse voltage, and the data were fitted to a negative Boltzmann equation of the form: I/I_max_ = 1/(1 + exp[(V_m_ − V_1/2_)/k)], where I is the current, I_max_ is the maximal current, V_1/2_ is the membrane potential for half-inactivation, V_m_ is the command potential, and k is the slope factor. Concentration-response curves were fitted using the following logistic equation: % inhibition = 100/[1+ (C/IC_50_)^*p*^], where C is the toxin concentration, *p* is the Hill coefficient (slope parameter), and IC_50_ is the median inhibitory concentration to inhibit mEPSC frequency.

## Electronic supplementary material

Discovery and identification of O, O-diethyl O-(4-(5-phenyl-4, 5-dihydroisoxazol-3-yl) phenyl) phosphorothioate (XP-1408) as a novel mode of action of organophosphorus insecticides
