# Supplementary material for: Discovery and identification of O, O-diethyl O-(4-(5-phenyl-4, 5-dihydroisoxazol-3-yl) phenyl) phosphorothioate (XP-1408) as a novel mode of action of organophosphorus insecticides
Source: Sci Rep. 2017 Jun 15;7:3617. doi: 10.1038/s41598-017-03663-3 (PMC5472594; doi:10.1038/s41598-017-03663-3)
Supplement: Supplementary file 1 — Discovery and identification of O, O-diethyl O-(4-(5-phenyl-4, 5-dihydroisoxazol-3-yl) phenyl) phosphorothioate (XP-1408) as a novel mode of action of organophosphorus insecticides [file 41598_2017_3663_MOESM1_ESM.pdf]

# Supporting Information for:

**Discovery and identification of *O*, *O*-diethyl *O*-(4-(5-phenyl-4, 5-dihydroisoxazol-3-yl) phenyl) phosphorothioate (XP-1408) as a novel mode of action of organophosphorus insecticides**

Zhigang Zeng[†, ‡, 1], Ying Yan[†, 1], Bingfeng Wang[†], Niu Liu[†], Hanhong Xu[†]*

[†] State Key Laboratory for Conservation and Utilization of Subtropical Agro-bioresources, Key Laboratory of Natural Pesticide and Chemical Biology of the Ministry of Education, South China Agricultural University, Guangzhou 510642, PR China

[†] School of Nuclear Technology and Chemistry & Biology, Hubei University of Science and Technology, Xianning 437100, PR China

**Corresponding Author**

*Hanhong Xu Tel: +86-20-85285127. E-mail: hhxu@scau.edu.cn

**Author Contributions**

[1]Zhigang Zeng and Ying Yan contributed equally to this work.

**Supporting Information Available**：

$^{1}$H NMR spectra of XP-1408· · · · · · · · · · · · · · · · · · · · Figure S1

$^{13}$C NMR spectra of XP-1408· · · · · · · · · · · · · · · · · · · · Figure S2

$^{31}$P NMR spectra of XP-1408· · · · · · · · · · · · · · · · · · · · Figure S3

HRMS spectra of XP-1408· · · · · · · · · · · · · · · · · · · · · Figure S4

The simulation result of Ach docking into the active site of AChE· · · · Figure S5

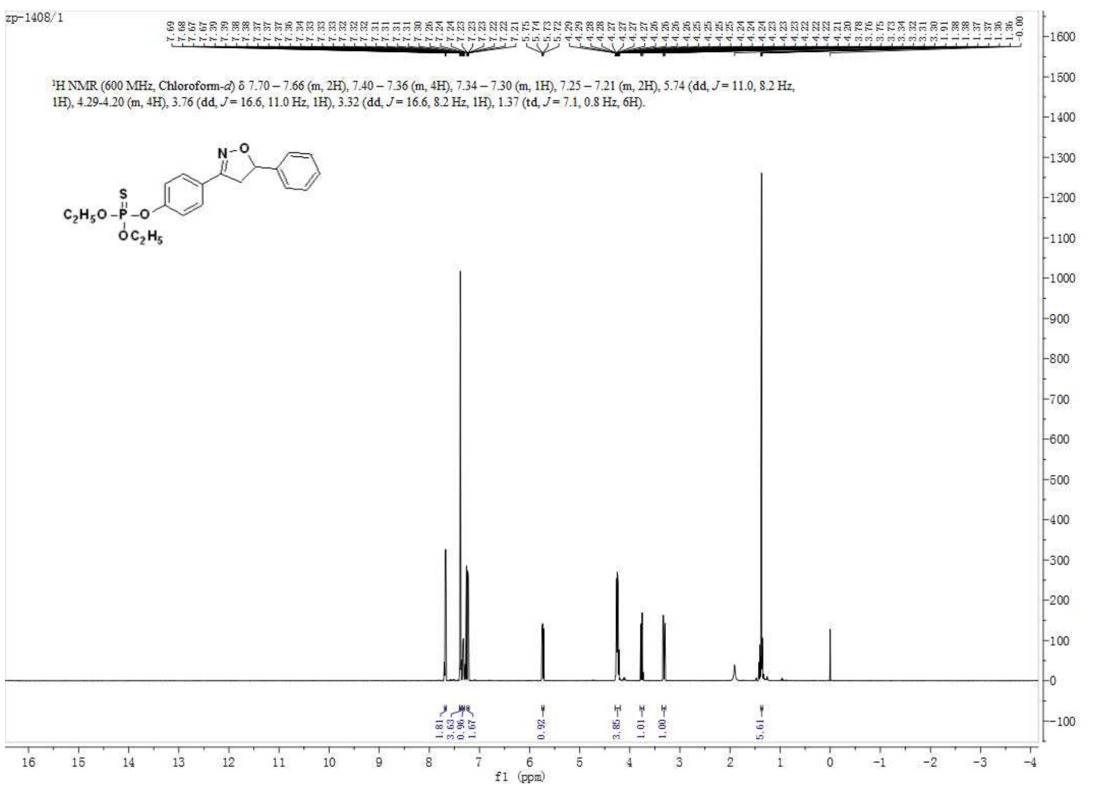


Figure S1. ¹H NMR spectra of XP-1408

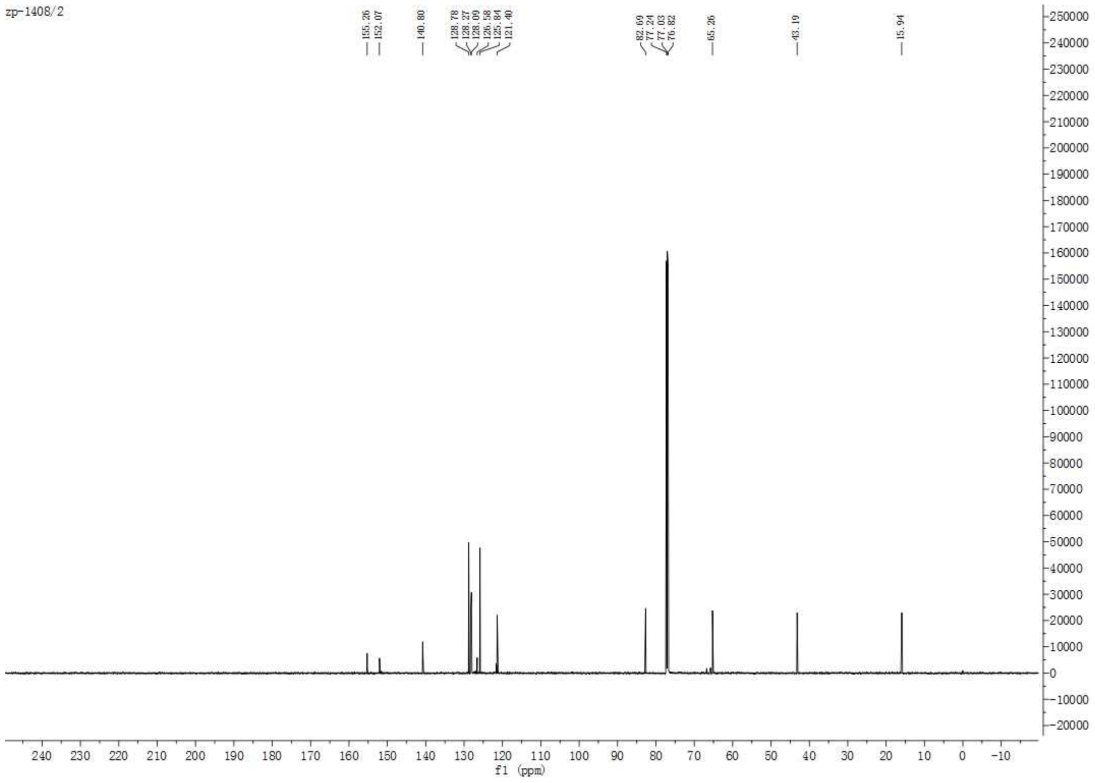


Figure S2. ¹³C NMR spectra of XP-1408

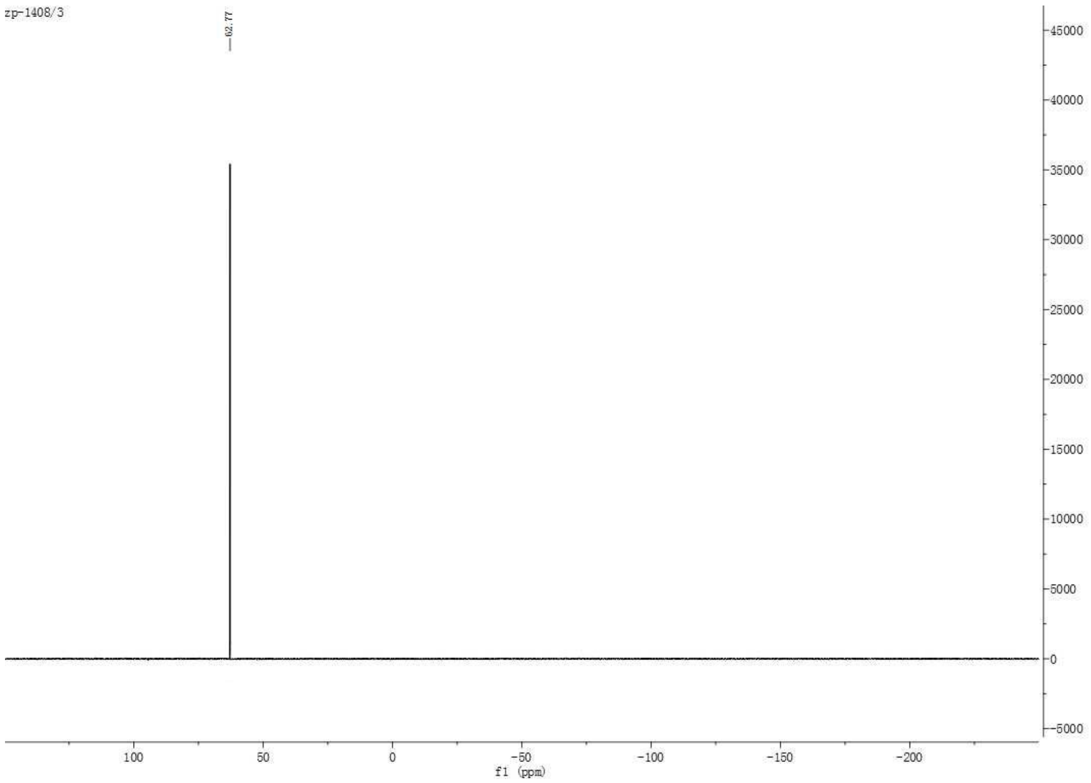


Figure S3. $^{31}$P NMR spectra of XP-1408

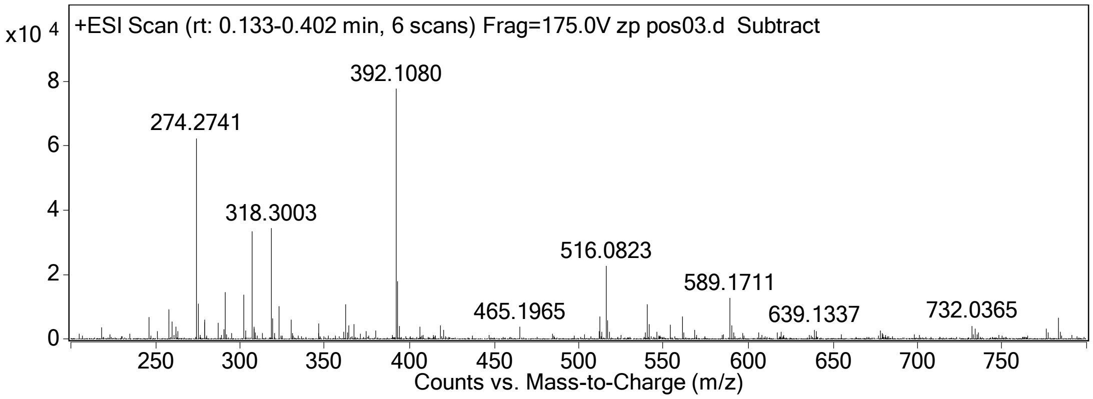


Figure S4. HRMS spectra of XP-1408

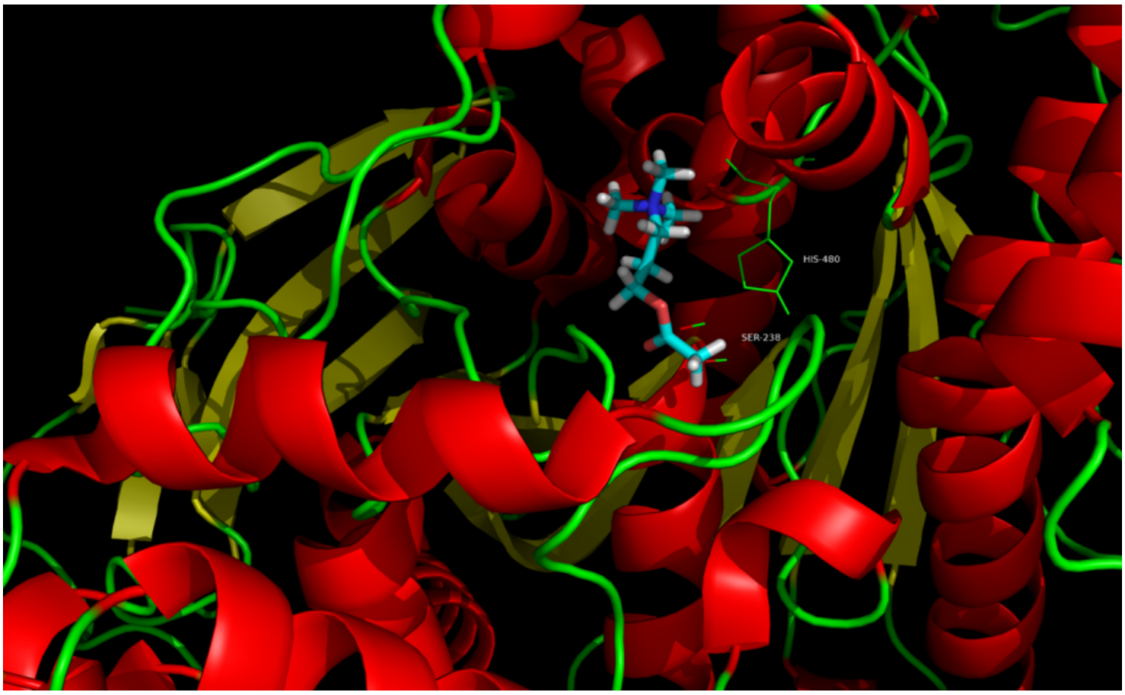


Figure S5. The simulation result of Ach docking into the active site of AChE
